# Trial-by-Trial Modulation of Associative Memory Formation by Reward Prediction Error and Reward Anticipation as Revealed by a Biologically Plausible Computational Model

**DOI:** 10.3389/fnhum.2017.00056

**Published:** 2017-02-15

**Authors:** Kristoffer C. Aberg, Julia Müller, Sophie Schwartz

**Affiliations:** ^1^Department of Neuroscience, Faculty of Medicine, University of GenevaGeneva, Switzerland; ^2^Swiss Center for Affective Sciences, University of GenevaGeneva, Switzerland; ^3^Geneva Neuroscience Center, University of GenevaGeneva, Switzerland

**Keywords:** associative memory, computational model, prediction error, reward anticipation, dopamine, personality trait, reward, punishment

## Abstract

Anticipation and delivery of rewards improves memory formation, but little effort has been made to disentangle their respective contributions to memory enhancement. Moreover, it has been suggested that the effects of reward on memory are mediated by dopaminergic influences on hippocampal plasticity. Yet, evidence linking memory improvements to actual reward computations reflected in the activity of the dopaminergic system, i.e., prediction errors and expected values, is scarce and inconclusive. For example, different previous studies reported that the magnitude of prediction errors during a reinforcement learning task was a positive, negative, or non-significant predictor of successfully encoding simultaneously presented images. Individual sensitivities to reward and punishment have been found to influence the activation of the dopaminergic reward system and could therefore help explain these seemingly discrepant results. Here, we used a novel associative memory task combined with computational modeling and showed independent effects of reward-delivery and reward-anticipation on memory. Strikingly, the computational approach revealed positive influences from both reward delivery, as mediated by prediction error magnitude, and reward anticipation, as mediated by magnitude of expected value, even in the absence of behavioral effects when analyzed using standard methods, i.e., by collapsing memory performance across trials within conditions. We additionally measured trait estimates of reward and punishment sensitivity and found that individuals with increased reward (vs. punishment) sensitivity had better memory for associations encoded during positive (vs. negative) prediction errors when tested after 20 min, but a negative trend when tested after 24 h. In conclusion, modeling trial-by-trial fluctuations in the magnitude of reward, as we did here for prediction errors and expected value computations, provides a comprehensive and biologically plausible description of the dynamic interplay between reward, dopamine, and associative memory formation. Our results also underline the importance of considering individual traits when assessing reward-related influences on memory.

## Introduction

Information entering the brain is varied and not equally well represented in memory. For example, aversive and rewarding events are usually better remembered as compared to neutral events (Kensinger, 2004; Maren and Quirk, 2004; Shohamy and Adcock, 2010). Improved memory for such events has an evolutionary advantage because it increases the chance of avoiding potential dangers as well as fulfilling crucial needs that typically engage reward processes, e.g., finding food and water, shelter, sexual partner. Although not yet fully understood, the neural mechanisms that enable such memory enhancement therefore promote survival.

Different aspects of reward have been reported to enhance memory. For example, recognition memory is enhanced for images serving as reward-predictive vs. neutral cues (Wittmann et al., 2005, 2011, 2013; Spaniol et al., 2014), suggesting a beneficial role of reward anticipation (Adcock et al., 2006). Memory is also promoted by the delivery of rewards, as indicated by enhanced recognition memory for items presented prior to positive vs. negative feedback (Mather and Schoeke, 2011) and high vs. low rewards (Murayama and Kitagami, 2014). Because both reward anticipation and reward delivery engages the dopaminergic reward system which projects to brain regions involved in memory processes, such as the hippocampus (Gabrieli, 1998; Squire et al., 2004), reward-related enhancements of memory may be determined by dopaminergic influences on hippocampal plasticity (Lisman and Grace, 2005; Shohamy and Adcock, 2010; Miendlarzewska et al., 2015). Some attempts have been made to disentangle the contribution of reward anticipation and reward delivery to memory formation. For example, Bialleck et al. (2011) used two categories of neutral objects as reward-predictive cues in a number comparison task, in which positive and negative outcomes were delivered. The authors tested two conditions, one where reward was contingent on task performance and one where it was not. In the reward-contingent block, i.e., the condition most similar to previous studies, recognition memory was found to be modulated by reward anticipation, but not reward delivery. By contrast, Mather and Schoeke (2011) reported a main effect of reward delivery on memory for pictures, but no effect of reward anticipation besides an interaction with image valence. In this latter study, no reward-learning occurred because pictures were presented as targets in a reaction time task and were preceded by reward-anticipatory cues with specific numbers (0, −0.25, +0.25$) indicating the value of the trial and followed by reward outcomes indicating hits or misses. The relationship between memory encoding, reward delivery, and reward anticipation is still unresolved.

However, because most previous studies averaged memory performance across trials within conditions of high or low rewards, significant fluctuations in reward computations associated with the dopamine system have been ignored. Specifically, rather than simply differentiating between positive and negative outcomes or between cues predicting rewards or neutral outcomes, dopamine neuron activity scales with the mismatch between actual and predicted outcomes, i.e., prediction errors (Schultz and Dickinson, 2000) and the magnitude of reward-predictive cue values, i.e., expected values (Tobler et al., 2005). These functional properties may have implications for the understanding of the links between reward, dopamine, and memory formation. For example, neutral objects may acquire reward-predictive values through the pairing with positive outcomes (Wittmann et al., 2005; Bialleck et al., 2011). This can be described by a process in which a cue's expected value is incremented following outcomes that are better than predicted, i.e., positive prediction errors, and decremented following outcomes that are worse than predicted, i.e., negative prediction errors (Rescorla and Wagner, 1972). Given initially neutral cues, learning typically develops gradually. In this case, because dopamine neuron activity tracks the magnitude of both expected values and prediction errors, a shift in dopamine activity occurs, whereby activity predominates at the time of reward delivery early during learning due to large prediction errors and small expected values, but is later observed at the time of cue presentation due to small prediction errors and large expected values (Schultz et al., 1997). Ignoring such fluctuations in reward makes it difficult to ascertain whether memory enhancing effects commonly attributed to reward anticipation are due to reward anticipation as a result of high expected values assigned to reward-predictive cues, reward delivery owing to large positive prediction errors, or both.

Two recent studies explicitly addressed the impact of reward computations on item recognition memory but reported seemingly discrepant results. Specifically, during a reinforcement learning task, Davidow et al. (2016) presented task-irrelevant images at reward delivery and found that adolescents, but not adults, showed increased recognition memory for images that had been presented during large prediction errors, when memory was tested immediately after the learning. By contrast, Wimmer et al. (2014) presented task-irrelevant images together with choice alternatives during a similar reinforcement learning task, and reported decreased recognition memory, in adult participants, for images presented in trials with large prediction errors, when memory was tested the next day. Potential explanations for these results may partly relate to the fact that Davidow et al. (2016) and Wimmer et al. (2014) tested memory immediately after learning and on the next day, respectively. Thus, the delay period between memory encoding and testing in Wimmer et al. (2014)'s study likely included a period of sleep, during which reward likely interacts with consolidation processes to promote memory formation (Lansink et al., 2009; Igloi et al., 2015). Moreover, differences between adolescents and adults may be mediated by increased reward-related activations of the dopaminergic reward system in adolescents (Galvan, 2010; Davidow et al., 2016). Thus, individual differences in the activation of the dopaminergic reward system, as determined by reward sensitive traits, may modulate the impact of reward during memory encoding. Yet, the impact of individual traits on reward-related modulations of episodic memory formation remains largely unknown.

**Figure 1:**
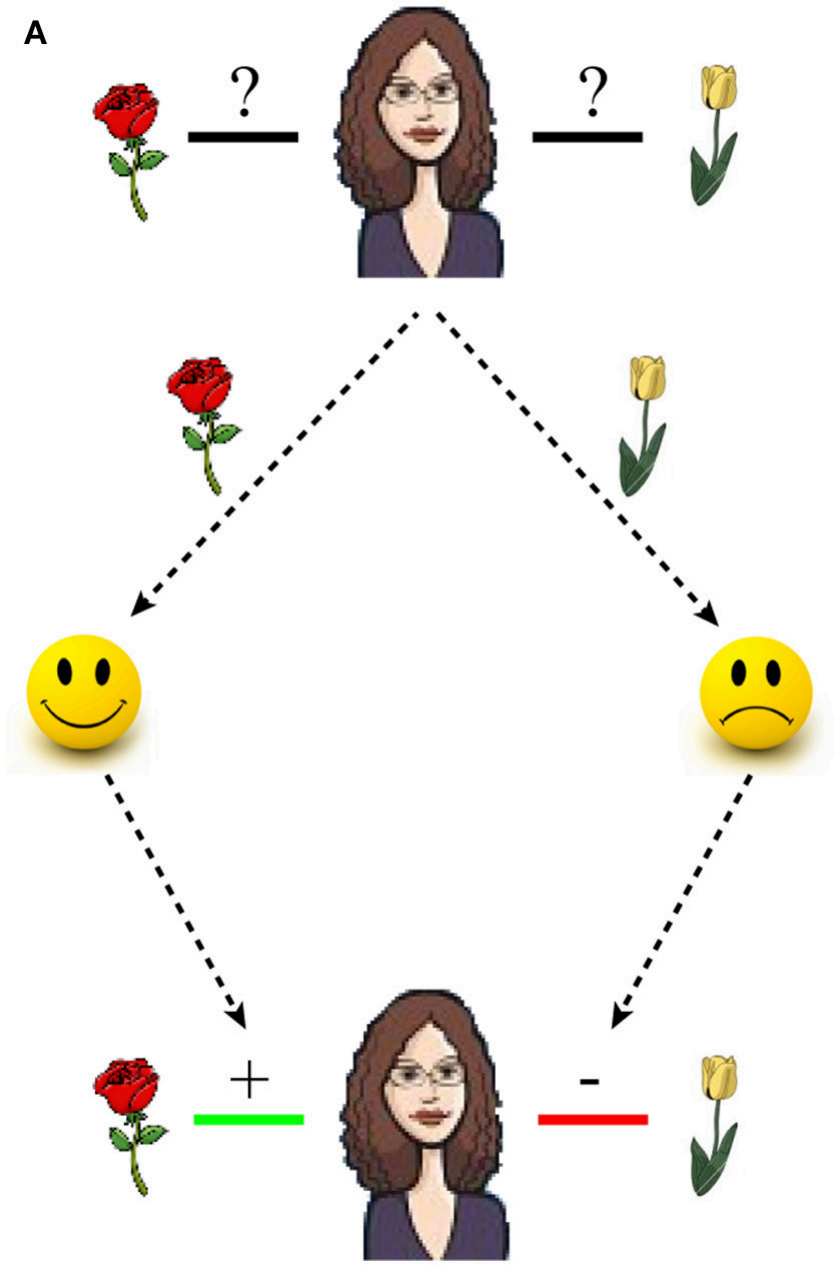
**Shaping associative memory via positive and negative feedback**. The problem of buying a spouse the right type of flowers becomes trivial by remembering the positive and negative associations between the spouse and each type of flower. In this example, a rose elicits a smile which causes a positive association while a tulip causes sadness, thus creating a negative association.

One final issue addressed by the present study is the current research bias toward using item recognition paradigms combined with monetary rewards. Recognition memory is just one facet of episodic memory and it seems unlikely that only monetary rewards should determine whether one memory is prioritized over others. For example, memorizing that a spouse prefers roses over tulips depends on the formation of associative memories, and such associations are shaped by aspects of social reward, such as the spouse's differential facial expression when receiving roses or tulips (see Figure 1). Social rewards evoke similar patterns of activity in the dopaminergic reward system as primary or monetary rewards. For example, presenting happy smiley faces increases activity in the ventral striatum, as compared to sad ones, and the mismatch between receiving a happy smiley face and the expectation of receiving it, i.e., the prediction error, is encoded in the dopaminergic midbrain (Aberg et al., 2015). Moreover, because associative memory formation depends on brain structures in the medial temporal lobe, including the hippocampus (Gold et al., 2006; Mayes et al., 2007), it is plausible to assume that dopaminergic influences on hippocampal plasticity also enhances associative memory formation.

To address these raised issues, the present study combines a novel associative memory task with a computational approach and estimates of trait reward and punishment sensitivity. In brief, during each encoding trial, participants learned character-object preferences by guessing which of two items a cartoon character preferred. Positive and negative feedback indicated whether selected objects were preferred or not preferred, and was also used to test the impact of reward delivery on memory encoding. Reward anticipation was experimentally manipulated by varying the ratio between the number of positive and negative feedbacks assigned to different characters. For example, characters assigned high reward anticipation received positive feedback in 8 out of 10 trials while those assigned to medium and low reward anticipation received positive feedback in 5/10 and 2/10 trials, respectively. Character-object associations were then tested after either 20 min or after 24 h. A computational approach incorporating computational characteristics of reward, namely trial-by-trial changes of expected value and prediction error, allowed us to decipher the respective contributions of reward anticipation and reward delivery on associative memory formation beyond simple averaging procedures. Individual differences in reward and punishment sensitivity was estimated via a French short version of the Sensitivity to Punishment and Sensitivity to Reward Questionnaire (SPSRQ).

## Materials and methods

### Participants

Twenty-six right-handed participants participated in the study. All participants provided written and informed consent according to the ethical regulations of the Geneva University Hospital and the study was performed in accordance with the Declaration of Helsinki. Data from one participant were excluded due to failing to adhere to task instructions, i.e., data from 25 participants were included in the statistical analyses (20 females; mean age ± SEM: 24.62 ± 1.08).

### Associative memory task

#### Encoding

**Figure 2:**
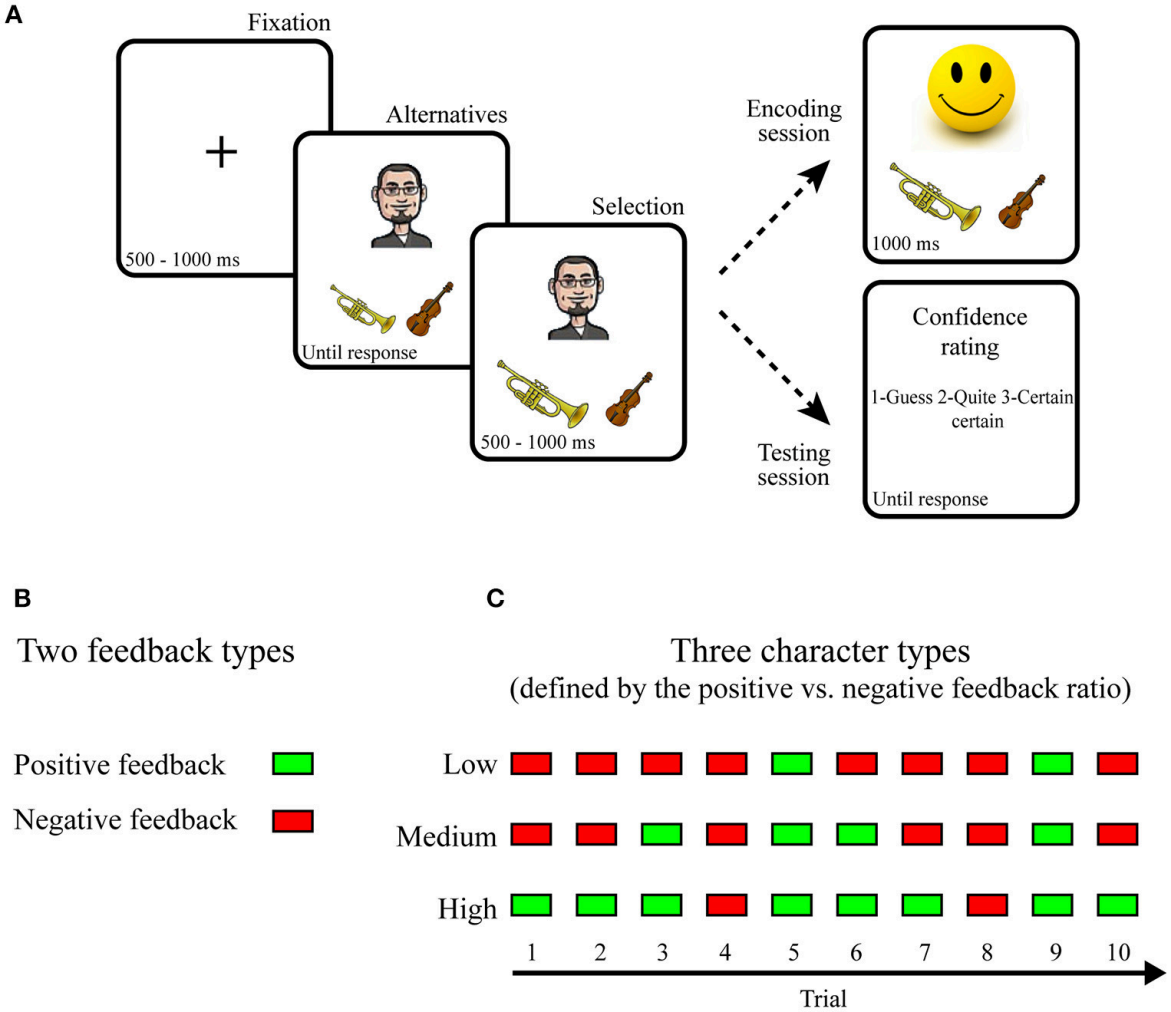
**Stimuli and Procedure. (A)** During encoding trials, a cartoon character was presented together with two objects. Participants guessed which of the two objects the character preferred and positive or negative feedback indicated whether the selected object was preferred or not preferred. During testing, trials were identical to the encoding with the exception that a confidence rating was performed instead of receiving feedback. **(B)** The impact of reward delivery on memory encoding was assessed through two types of feedback, positive and negative. **(C)** The impact of reward anticipation on memory encoding was assessed by manipulating the ratio between the number of positive and negative feedback assigned to a character. Specifically, characters were divided into three different categories.

Each trial started with the presentation of a fixation cross, after which the face of a cartoon character was presented together with two objects (Figure 2A). Participants were instructed that the character preferred one object in each pair and they had to guess the preferred object by pressing a left or a right button with their right hand. Following selection, a positive feedback (happy smiley face) or negative feedback (sad smiley face) indicated whether the participant had guessed the preferred or the non-preferred object. Participants were instructed that they would later be tested on the preferences. Positive and negative feedbacks were therefore equally valid in terms of learning to discriminate between the preferred and the non-preferred object in each pair.

Each pair of objects was presented once for each character and participants encoded 10 preferences for each character before encoding 10 preferences for the next character, and so forth. In total, participants learned 10 character-object preferences for six different characters, resulting in a total of 60 trials.

In accordance with standard procedures, memory between different conditions of reward-delivery and anticipation was tested by collapsing memory performance across trials within a condition. Specifically, the influence of reward delivery was tested by comparing average memory performance for trials with positive and negative feedbacks (Figure 2B), while the impact of reward anticipation was assessed by comparing average memory performance for characters assigned to different feedback ratios (Figure 2C). The feedback ratios were manipulated in a pre-determined fashion such that the feedback presented in a trial did not depend on the actual selection. For example, for characters assigned to high reward anticipation positive feedback was presented in 8/10 trials, while feedback was positive in 5/10 and 2/10 trials for characters assigned to medium and low reward anticipation (Figure 2C). In total, two characters were assigned to each of three levels of reward anticipation (high, medium, and low; Figure 2C). Moreover, because the feedback ratios were determined probabilistically, the reward anticipatory value of each character fluctuated from trial to trial. This allowed studying how subtle fluctuations in reward, as estimated by a computational model (see Section Computational approach below), influenced associative memory formation.

#### Testing

During testing, the procedure was identical to the training with the exception that feedback was no longer provided (Figure 2A). Participants were instructed to select the preferred object in each pair. This could be accomplished by recalling and selecting the preferred object, or by recalling and rejecting the non-preferred object. Instead of receiving feedback, participants rated the confidence of their selection as “a Guess,” “Quite certain,” or “Certain.”

#### Procedure

To determine whether reward-influences on memory formation were present after short (Bialleck et al., 2011; Mather and Schoeke, 2011; Davidow et al., 2016) and/or long over-night delays between encoding and recall (Wittmann et al., 2005, 2011, 2013; Murayama and Kitagami, 2014; Wimmer et al., 2014), each participant performed two encoding-testing sessions. Participants in one group (*n* = 13, 13 females; mean age ± SEM: 25.44 ± 0.94) first encoded character-object preferences and then performed an unrelated visual discrimination task (lasting approximately 20 min) after which memory for the preferences were tested. After a break new character-object preferences were encoded followed by the unrelated visual discrimination task, but memory for these new preferences was tested 24 h later. Another group of participants (*n* = 12, 7 females; mean age ± SEM: 23.79 ± 1.27) performed the encoding-testing sessions in reverse order, i.e., they first performed the session with a 24 h delay between encoding and recall, and then the one with a 20 min delay. Of note, neither feedback nor reward was provided during the visual task to avoid any interference with associative memory formation. Moreover, new characters and pairs were presented in each session to prevent memory interference between sessions. Before the experiment, participants performed a training version of the task to get familiarized with the experimental paradigm. Six character-item preferences were encoded (three with positive and three with negative feedback) and tested after a brief break. The characters and the object-pairs used for the training were not included in the experiment proper.

### Statistical analyses

#### Behavior

We first used standard procedures in the field, i.e., averaging performance across trials within each condition, to assess the impact of reward anticipation and delivery on memory. We thus calculated the proportion of correct selections/rejections for each condition (Character types; Feedback types). Differences between conditions were analyzed using ANOVAs and Monte-Carlo permutation tests (MC-tests). MC-tests are less sensitive to violations of normality and are therefore more suitable for small sample sizes as compared to the traditional *t*-test (Howell, 2013). We then applied a computational approach in order to account for trial-by-trial fluctuations in memory formation as a function of reward delivery and reward anticipation, as described below.

#### Computational approach

Reward-related enhancements of memory formation have been attributed to dopaminergic influences on hippocampal plasticity (Wittmann et al., 2005; Adcock et al., 2006; Shohamy and Adcock, 2010). Two specific aspects of reward are known to scale with dopamine neuron activity, i.e., expected value (the level of anticipated reward) and prediction error (the mismatch between actual and predicted outcomes). Specifically, phasic dopamine neuron activity increases when expected values increase (Tobler et al., 2005) and when outcomes are better (more positive) than predicted (Schultz et al., 1997). Thus, in an attempt to elucidate the relationship between specific reward computations and associative memory formation, expected values and prediction errors were incorporated into a novel computational model of associative memory.

Each character type in the present study is defined through its repeated associations with positive and negative feedback, with the underlying assumption that different feedback ratios will yield different expected values. This learning process can be described by a Q-learning rule (Watkins and Dayan, 1992). In each trial *t*, the expected value *V*_*c*_ of character *c* is updated based on the prediction error δ, i.e., the mismatch between the actual outcome *r* (here set to 1 and 0 for positive and negative outcomes, respectively) and the expected value *V*_*c*_, scaled by the learning rate α:

$$\begin{array}{l} \delta \left(t\right)=r\left(t\right)-V_{c}\left(t\right)\\ V_{c}\left(t+1\right)=V_{c}\left(t\right)+\alpha \cdot \delta \left(t\right) \end{array}$$

These concepts were then incorporated into an associative memory model which assumes that the probability of encoding a character-object association depends on the reward *R*(*t*) provided in each trial *t*, i.e., the magnitude of the expected value *V*_*c*_(*t*) and the prediction error δ(*t*), as well as a constant *C*_0_. The probability *p*_*Memory*_(*t*) of encoding information presented in a trial *t* is described through a logistic function:

$$\begin{array}{l} p_{Memory}\left(t\right)=\frac{1}{1+e^{-R\left(t\right)}} \end{array}$$

To determine which aspect of reward that contributes to memory formation, different models were confronted:

A “Baseline” model without any reward contribution: *R*(*t*) = *C*_0_
A “δ” model with a contribution of only signed prediction error: *R*(*t*) = *C*_0_ + *C*_δ_ · δ(*t*)
A “V” model with a contribution of only expected value: *R*(*t*) = *C*_0_ + *C*_*V*_ · *V*_*c*_(*t*)
A “δ+V” model with contributions from signed prediction error and expected value: *R*(*t*) = *C*_0_ + *C*_*V*_ · *V*_*c*_(*t*) + *C*_δ_ · δ(*t*)

One reviewer pointed out the involvement of the noradrenergic system in both memory formation and the encoding of “surprise,” i.e., unsigned prediction errors. Potential noradrenergic contributions to memory formation were therefore tested in two additional models:

A “|δ|” model with a contribution of only unsigned prediction error: *R*(*t*) = *C*_0_ + *C*_|δ|_ · |δ(*t*)|
A “|δ|+V” model with contributions from unsigned prediction error and expected value: *R*(*t*) = *C*_0_ + *C*_*V*_ · *V*_*c*_(*t*)+*C*_|δ|_ · |δ(*t*)|

*C*_*V*_, *C*_δ_, and *C*_|δ|_ are scale factors that determine the respective contribution of expected value, signed prediction error, and unsigned prediction error to the memory encoding probability. The free parameters *C*_0_, *C*_*V*_, *C*_δ_, *C*_|δ|_, and α were fitted to each participant's data through maximum likelihood estimation, i.e., by minimizing the negative log-likelihood estimation function (LLE) for logistic regression:

$$\begin{array}{lll} LLE & = & -\overset{n}{\underset{t\;=\;1}{\sum }}y\left(t\right)\;*\;log\;p_{Memory}\left(t\right)+\left(1-y\left(t\right)\right)\\ {}*\;log\;\left(1-p_{Memory}\left(t\right)\right) \end{array}$$

*y*(*t*) is the observed outcome (i.e., hit/miss) in each trial *t*. Minimization of the log-likelihood was performed in two steps. First, for each model the LLE was calculated for the complete parameter space in steps of 0.01, i.e., −2:0.01:2 for all scale factors *C*_*x*_ and 0:0.01:1 for α. A more refined search was then performed via a Nelder-Mead simplex method (Nelder and Mead, 1965), in which the parameter search space was centered on the optimal values obtained from the first step with boundaries set to ±0.01 around these values.

Model fits were compared using the Akaike Information Criterion (AIC; Akaike, 1974) which accounts for different numbers of fitted variables *k*:

$$\begin{array}{l} AIC=2\;*\;k+2\;*\;LLE \end{array}$$

### Questionnaires

Individual differences in the sensitivity to reward and punishment modulate the balance between approach and avoidance learning (Smillie et al., 2007; Aberg et al., 2016). To investigate whether such trait characteristics also influence associative memory formation, we administered a questionnaire estimating reward and punishment sensitivity, i.e., a French version of the Sensitivity to Punishment and Sensitivity to Reward Questionnaire (SPSRQ; Lardi et al., 2008) previously shown to be related to the balance between learning from positive (vs. negative) feedback in a reinforcement learning task (Aberg et al., 2016). Six of the participants were unable to fill out the questionnaires because they were not native French speakers, thus these data were collected on 19 out of the 25 participants. Correlations between memory performance and traits were investigated using the Spearman rank-correlation coefficient ρ. A sensitivity bias score was calculated as the z-scored Sensitivity to Reward minus the z-scored Sensitivity to Punishment, i.e., z-score SR-SP (Aberg et al., 2016).

## Results

### Memory performance

An initial ANOVA on memory performance did not reveal any main effect of Group (participants first tested after 20 min vs. first tested after 24 h) or significant two-, three-, four-, or five-way interactions between the factor Group and factors Feedback type (positive, negative feedback), Character type (low, medium, high positive vs. negative feedback ratio), Confidence level (guess, quite certain, certain), or Delay (20 min, 24 h; all *p* > 0.1). Thus, the factor Group was removed from all subsequent analyses.

#### Memory tested after 20 min

**Figure 3:**
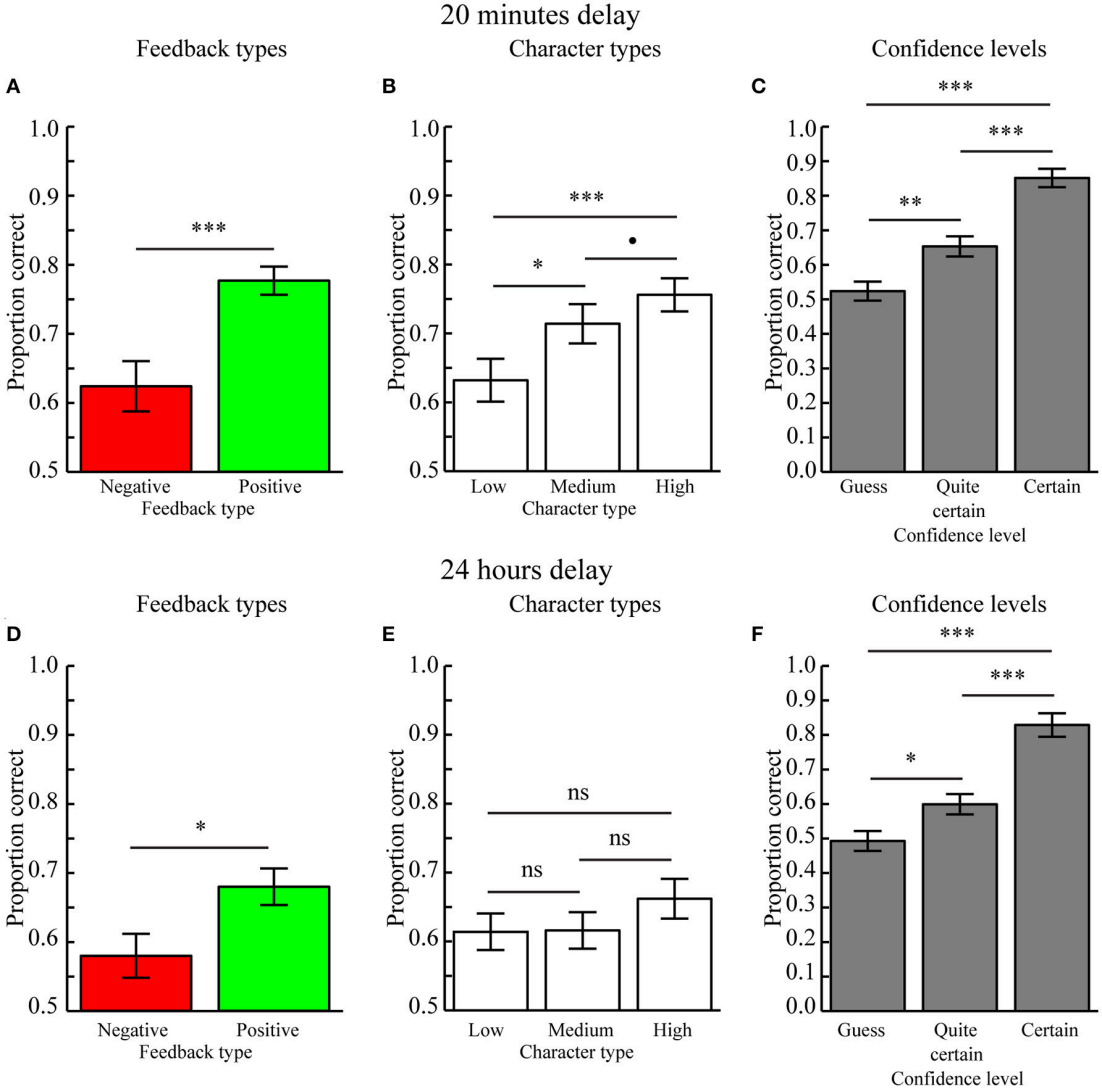
**Behavioral results (Mean ± SEM). (A)** Main effect of Feedback type when tested after 20 min. Memory performance was higher for character-item associations encoded with positive as compared to negative feedback. **(B)** Main effect of Character type when tested after 20 min. Memory performance for character-object associations scaled with the positive vs. negative feedback ratio. **(C)** Main effect of Confidence level when tested after 20 min. Performance was highest when the memory for character-item associations was rated as Certain, as compared to those rated as Quite certain, which were better recalled than Guesses. **(D)** Main effect of Feedback type when tested after 24 h. Memory performance was higher for character-object associations encoded with positive feedback as compared to negative feedback. **(E)** Memory performance did not differ between Character types when tested after 24 h. **(F)** Main effect of Confidence level when tested after 24 h. Performance was highest when the memory for character-item associations was rated as Certain, as compared to those rated as Quite certain, which were better recalled than Guesses. ^•^*p* < 0.1, ^*^p < 0.05, ^**^p < 0.01, ^***^p < 0.001, ns = not significant (*p* > 0.05).

A three-way repeated measures ANOVA with factors Feedback type (positive, negative feedback), Character type (high, medium, low feedback ration), and Confidence level (guess, quite certain, certain) revealed a main effect of Feedback type [*F*_(1, 24)_ = 21.345, *p* = 0.004] because character-object preferences encoded during positive feedback (mean ± SEM: 0.777 ± 0.020) were better remembered as compared to those encoded during negative feedback (mean ± SEM: 0.624 ± 0.036, *p* < 0.001; Figure 3A). Moreover, there was a trend for an effect of Character type [*F*_(2, 48)_ = 3.975, *p* = 0.061] because character-object preferences encoded for characters associated with a high feedback ratio (mean ± SEM: 0.756 ± 0.024) were better remembered as compared to those associated with a low feedback ratio (mean ± SEM: 0.632 ± 0.031, *p* < 0.001) and marginally better as compared to those associated with a medium feedback ratio (mean ± SEM: 0.714 ± 0.029, *p* = 0.098). Memory performance between characters associated with medium and low feedback ratios was also significant (*p* = 0.018; Figure 3B). There was also a significant main effect of Confidence level [*F*_(2, 48)_ = 44.348, *p* < 0.001] because memory performance was higher for Certain (mean ± SEM: 0.852 ± 0.027) as compared to Quite certain responses (mean ± SEM: 0.653 ± 0.029, *p* < 0.001) and Guesses (mean ± SEM: 0.524 ± 0.028, *p* < 0.001). Memory performance for Quite certain responses was also better as compared to Guesses (*p* = 0.001) (Figure 3C). No two- or three-way interactions were significant (all *p* > 0.13), therefore suggesting independent influences of reward anticipation (Character type) and reward delivery (Feedback type) on memory formation. In addition, this means there was no reward-modulation of the relationship between subjective confidence levels and memory performance.

The effect of Character type was confirmed in a subsequent analysis where the linear regression slopes between memory performance and character types were calculated separately for positive and negative feedback. A paired MC-test revealed that the slopes did not differ between positive feedback (mean ± SEM: 0.039 ± 0.026) and negative feedback (mean ± SEM: 0.028 ± 0.022, *p* = 0.762). However, the average slope across feedback types (mean ± SEM: 0.062 ± 0.016) was significantly different from 0.0 (*p* < 0.001), indicating a significant overall effect of Character type on memory performance.

#### Memory tested after 24 h

A three-way repeated measures ANOVA with factors Feedback type (positive, negative feedback), Character type (high, medium, low feedback ratio), and Confidence level (guess, quite certain, certain) revealed no main effect of Feedback type [*F*_(1, 24)_ = 1.933, *p* = 0.178], although a paired MC-test revealed that associations encoded during positive feedback (mean ± SEM: 0.680 ± 0.027) was significantly better as compared to those encoded during negative feedback (mean ± SEM: 0.580 ± 0.032, *p* = 0.022; Figure 3D). The effect of Character type was not significant [*F*_(2, 48)_ = 0.394, *p* = 0.677], as confirmed by paired MC-tests, i.e., high (mean ± SEM: 0.662 ± 0.029) vs. low (mean ± SEM: 0.614 ± 0.027, *p* = 0.212), high vs. medium (mean ± SEM: 0.616 ± 0.027, *p* = 0.156), and low vs. medium feedback ratios (p = 0.973; Figure 3E). The main effect of Confidence level was significant [*F*_(2, 48)_ = 19.709, *p* < 0.001) because memory performance was higher for Certain (mean ± SEM: 0.829 ± 0.034) as compared to Quite certain responses (mean ± SEM: 0.599 ± 0.029, *p* < 0.001) and Guesses (mean ± SEM: 0.493 ± 0.029, *p* < 0.001). Memory performance for Quite certain responses was also better as compared to Guesses (*p* = 0.019) (Figure 3F). There were no significant two- or three-way interactions (all *p* > 0.644). Moreover, there was no difference in the regression slopes relating memory performance to different Character types for positive feedback (mean ± SEM: −0.003 ± 0.028) as compared to negative feedback (mean ± SEM: 0.006 ± 0.027, *p* = 0.738). The regression slopes calculated across feedback types (mean ± SEM: 0.024 ± 0.018) were also not significantly different from 0.0 (*p* = 0.223).

As when memory was tested after 20 min, memory performance was graded by confidence levels, i.e., Certain > Quite certain > Guess, also when tested after 24 h. Together these results replicate the robust relationship between confidence levels and memory accuracy (De Zubicaray et al., 2011; Qin et al., 2011; Kuchinke et al., 2013). Because there were no significant interactions between Confidence levels and the Feedback or Character types when memory was tested after 20 min or after 24 h, these results are not discussed any further.

### Computational approach

To explain these behavioral results in light of specific reward computations associated with the dopaminergic system, a computational approach was applied. Because previous studies indicate influences of both reward anticipation and delivery of rewards on memory formation, and because dopamine activity tracks the magnitude of reward anticipation, i.e., expected value, and reward delivery, i.e., prediction errors, we predicted that a model combining these two concepts would provide the best fit to behavioral data. Moreover, to confirm whether expected values and/or prediction errors contributed significantly to the memory formation, the values of the fitted scale factors of the best fitting model were compared to the null hypothesis of no influence, i.e., that their respective average value is equal to 0.

#### Memory tested after 20 min

**Figure 4:**
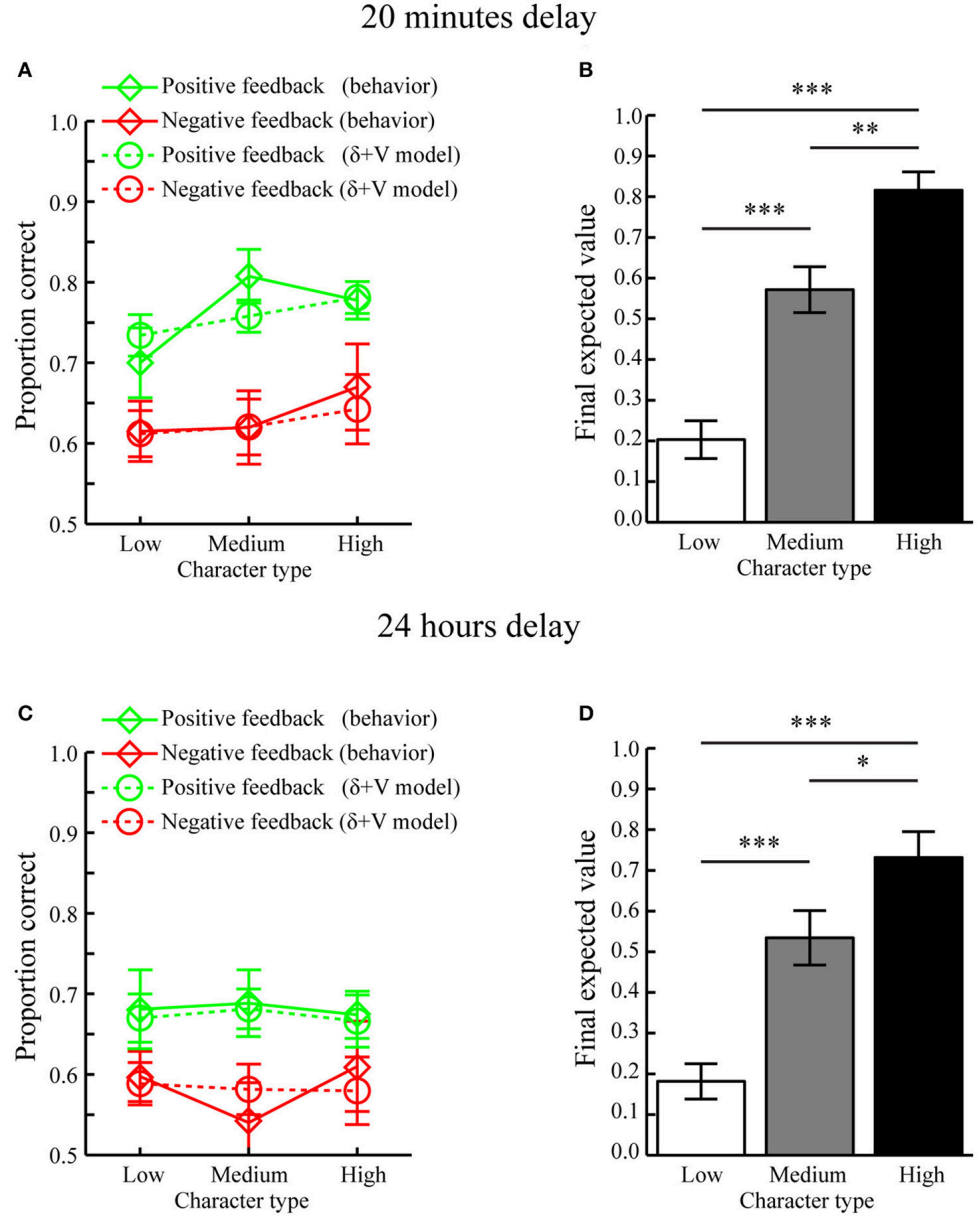
**Computational modeling results (Mean ± SEM). (A)** Memory performance as a function of Feedback and Character types when tested after 20 min. Actual behavior is shown as solid lines while predictions of the best fitting model, i.e., the δ+V model, are displayed as dotted lines. **(B)** Average final expected value for different character types when tested after a 20 min delay. **(C)** Memory performance as a function of Feedback and Character types when tested after 24 h. Predictions of the best fitting model, i.e., the δ+V model, are displayed as dotted lines. **(D)** Average final expected value for different character types when tested after a 24 h delay. ^*^p < 0.05, ^**^p < 0.01, ^***^p < 0.001.

Average fitted parameters and AIC values for the different models are displayed in Table 1. Comparing AIC values using paired MC-tests revealed that the “δ+V” model, incorporating contributions from signed prediction errors (δ) and expected values (V), provided the best fit as compared to the other models (δ+V vs. Baseline, *p* < 0.001; δ+V vs. δ, *p* = 0.011; δ+V vs. V, *p* = 0.005; δ+V vs. |δ|, *p* < 0.016; δ+V vs. |δ|+V, *p* < 0.020). The fit of the δ+V model to behavioral data is shown in Figure 4A (dashed lines).

**Table 1:** **Model fits**.

				**Scale factors**			
**Model**	**Model fit (LLE)**	**Corrected model fit (AIC)**	**Offset (C_0)_**	**Signed prediction error (C_δ_)**	**Unsigned prediction error (C_|δ|_)**	**Expected value (C*_V_*)**	**Learning rate (α)**
**20 MIN DELAY**							
Baseline (no reward influence)	70.755 ± 1.928	143.510 ± 3.856	0.721 ± 0.075	–	–	–	–
Signed prediction error (δ)	60.440 ± 1.636	126.881 ± 3.272	0.735 ± 0.071	0.369 ± 0.158	–	–	0.395 ± 0.077
Unsigned prediction error (|δ|)	61.718 ± 1.989	129.436 ± 3.979	0.562 ± 0.107	–	0.503 ± 0.166	–	0.583 ± 0.074
Expected value (V)	61.593 ± 2.124	129.186 ± 4.247	0.584 ± 0.104	–	–	0.501 ± 0.221	0.372 ± 0.064
Signed prediction error and expected value (δ+V)^*^	57.828 ± 1.978	123.656 ± 3.957	0.350 ± 0.132	0.725 ± 0.154	–	1.156 ± 0.180	0.329 ± 0.057
Unsigned prediction error and expected value (|δ|+V)	59.625 ± 2.143	127.250 ± 4.287	0.530 ± 0.099	–	0.361 ± 0.212	0.360 ± 0.239	0.456 ± 0.075
**24 H DELAY**							
Baseline (no reward influence)	76.445 ± 1.723	154.889 ± 3.447	0.496± 0.026	–	–	–	–
Signed prediction error (δ)	64.079 ± 1.625	134.159 ± 3.250	0.507 ± 0.073	0.215 ± 0.171	–		0.333 ± 0.068
Unsigned prediction error (|δ|)	65.270 ± 2.201	136.539 ± 4.401	0.417 ± 0.108	–	0.292 ± 0.202	–	0.404 ± 0.085
Expected value (V)	64.267 ± 1.968	134.535 ± 3.937	0.402 ± 0.117	–	–	0.268 ± 0.265	0.456 ± 0.080
Signed prediction error and expected value (δ+V)^*^	61.165 ± 1.985	130.329 ± 3.970	0.223 ± 0.123	0.469 ± 0.173	–	0.729 ± 0.274	0.312 ± 0.066
Unsigned prediction error and expected value (|δ|+V)	62.943 ± 2.291	133.887 ± 4.582	0.284 ± 0.146	–	0.327 ± 0.199	0.277 ± 0.257	0.433 ± 0.078

LLE is the negative log-likelihood estimate. AIC is Akaike's Information Criterion. The scale factors C_δ_, C_|δ|_, and C_V_ determine the contribution of signed prediction errors, unsigned prediction errors, and expected value to memory performance, respectively. α is the learning rate for the Q-learning rule.

* *Denotes the model providing the best fit to behavioral data, as indicated by significantly lower AIC values (see Section Computational Approach). Mean ± SEM*.

To confirm that reward-learning had occurred, the final expected values of each character type, as estimated through the “δ+V” model, are displayed in Figure 4B. Paired MC-tests revealed that characters associated with high reward anticipation (i.e., high feedback ratios) had obtained a higher final expected value (mean ± SEM: 0.816 ± 0.045) as compared to characters with medium (mean ± SEM: 0.575 ± 0.056) and low (mean ± SEM: 0.203 ± 0.046) reward anticipation, who also differed between each other (all *p* < 0.006).

Next, it was tested whether the average values of the fitted scale factors *C*_*V*_ and *C*_δ_ for the “δ+V” model (see Table 1) were significantly larger than 0. Indeed, MC-tests revealed that both *C*_*V*_ (*p* < 0.001) and *C*_δ_ (*p* < 0.001) were significantly larger than 0.0. Because *C*_*V*_ and *C*_δ_ index the contribution of expected value and signed prediction error magnitudes to memory performance, both of these reward computations contributed to subsequent memory formation when tested after 20 min.

#### Memory tested after 24 h

Average fitted parameters and AIC values are displayed in Table 1. Similar to when tested after 20 min, paired MC-tests showed that the “δ+V” model provided the best fit to behavioral data (δ+V vs. Baseline, *p* < 0.001; δ+V vs. δ, *p* = 0.001, δ+V vs. *V, p* = 0.014; δ+V vs. |δ|, *p* < 0.023; δ+V vs. |δ|+V, *p* < 0.046). The fit of the “δ+V” model to behavioral data is shown in Figure 4C.

The expected values of each character type, as estimated through the “δ+V” model, are displayed in Figure 4D. Paired MC-tests revealed that expected values for characters associated with high reward anticipation had obtained a higher final expected value (mean ± SEM: 0.732 ± 0.064) as compared to characters with medium (mean ± SEM: 0.534 ± 0.067) and low (mean ± SEM: 0.181 ± 0.043) reward anticipation, who also differed between each other (all *p* < 0.032).

Moreover, the average values for *C*_*V*_ (*p* < 0.013) and *C*_δ_ (*p* < 0.017) for the “δ+V” model (see Table 1) were significantly larger than 0.0. Importantly, these results indicate that both expected value and prediction error contributed to memory formation when tested after 24 h, despite no significant effect of reward anticipation when memory performance was averaged across trials (i.e. Character type; Section Memory performance). Thus, unlike standard analyses, which typically average memory performance across trials within conditions, our computational approach, which incorporates trial-by-trial fluctuations in the magnitude of reward during encoding, was able to clarify how reward significantly influenced memory formation also when tested after 24 h. The model is therefore not only more sensitive but, because it integrates existing physiological constraints to study how reward affects behavior, it presumably provides a more accurate description of how reward influences memory formation.

### Effects of individual sensitivity to reward/punishment on memory

Individual differences in the sensitivity to reward and punishment are reflected in the responsiveness of the dopaminergic reward system (Simon et al., 2010; Kennis et al., 2013), and bias the ability to learn from positive and negative outcomes (Aberg et al., 2016). To test how biases in the expression of individual traits impact on reward-related memory enhancements, individual memory performance was correlated with the differential score between the Sensitivity to Reward and the Sensitivity to Punishment scales of the SPSRQ.

#### Memory tested after 20 min

**Figure 5:**
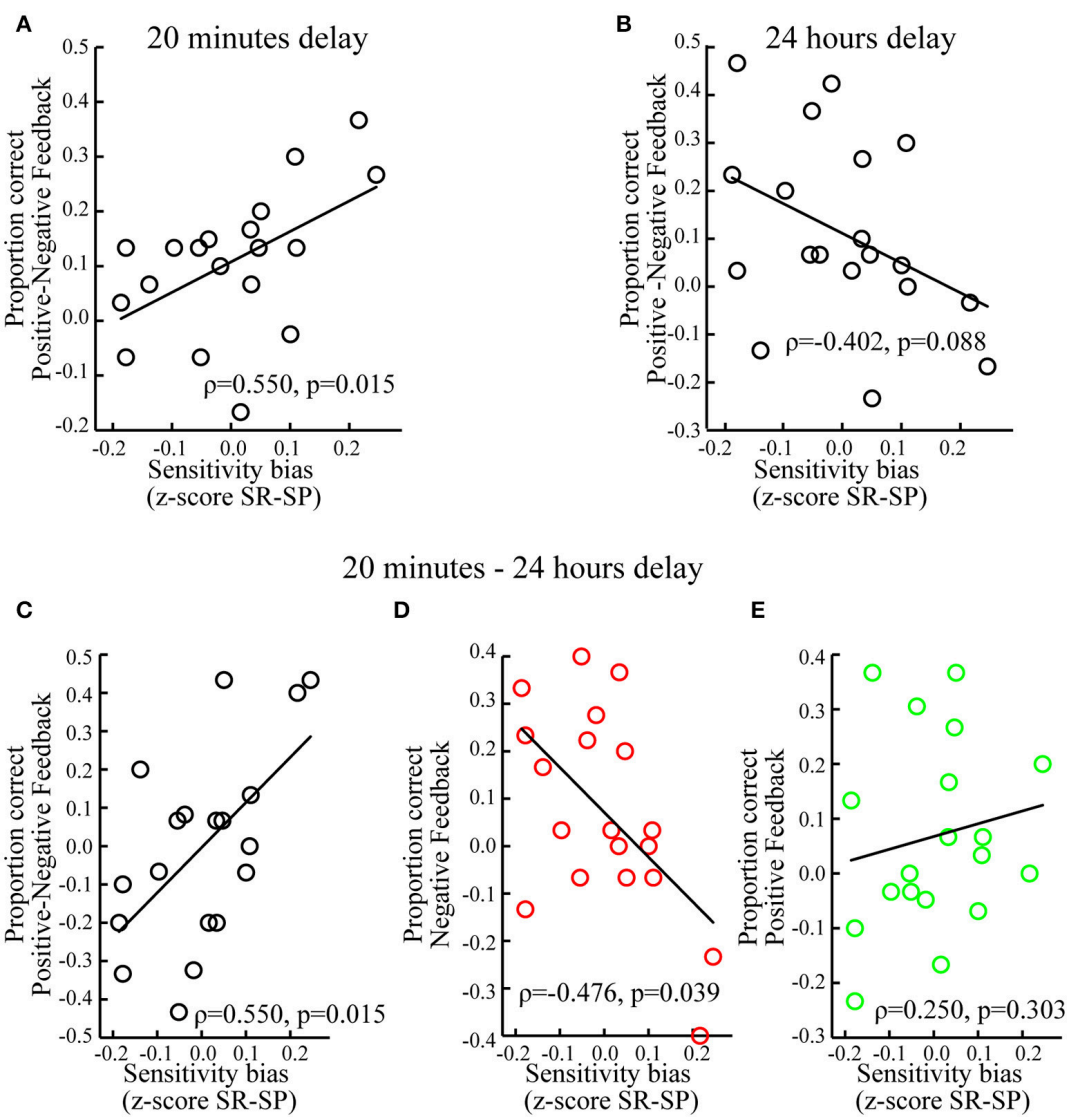
**Individual differences. (A)** When tested after 20 min, memory performance for positive vs. negative feedback correlated positively with the sensitivity bias, i.e., z-scored Sensitivity to Reward (SR) minus z-scored Sensitivity to Punishment (SP). **(B)** When tested after 24 h, memory performance for positive vs. negative feedback correlated marginally and negatively with the sensitivity bias. **(C)** For positive vs. negative feedback, memory performance correlated positively with the sensitivity bias, when memory was tested after 20 min as compared to 24 h. **(D)** For negative feedback, memory performance correlated negatively with the sensitivity bias, when memory was tested after 20 min as compared to 24 h. **(E)** For positive feedback, memory performance did not correlate significantly with the sensitivity bias, when memory was tested after 20 min as compared to 24 h. For display purposes non-ranked data and linear regression slopes are shown.

Individuals with a sensitivity bias favoring reward over punishment showed better memory for associations encoded during positive (vs. negative) feedback (ρ = 0.550, *p* = 0.015; Figure 5A). Looking at positive and negative feedback separately revealed no significant correlations between the sensitivity bias and memory associated with positive (ρ = 0.209, *p* = 0.391) or negative feedback (ρ = −0.326, *p* = 0.173). Testing whether individual sensitivity biases predicted to what extent memory performance was influenced by prediction errors, i.e., *C*_δ_, and expected values, i.e., *C*_*V*_, showed a significant correlation between the sensitivity bias and *C*_δ_ (ρ = 0.457, *p* = 0.049) but not with *C*_*V*_ (ρ = 0.008, *p* = 0.974).

#### Memory tested after 24 h

There was a marginally significant negative correlation between the sensitivity bias and memory for associations encoded during positive (vs. negative) feedback (ρ = −0.402, *p* = 0.088; Figure 5B). This tendency was mostly driven by associations encoded during negative feedback (ρ = 0.415, *p* = 0.077), but not positive feedback (ρ = −0.167, *p* = 0.496). The sensitivity bias did not correlate with *C*_δ_ (ρ = −0.109, *p* = 0.657) or *C*_*V*_ (ρ = −0.134, *p* = 0.584).

#### Difference between memory tested after 20 min and 24 h

Finally, we tested the influence of the sensitivity bias on the differential memory performance between short and long delays, i.e., memory tested after 20 min (vs. 24 h). An increased expression of reward (vs. punishment) sensitivity was associated with relatively better memory for associations encoded during positive (vs. negative) feedback when tested after 20 min as compared to 24 h (ρ = 0.550, *p* = 0.015; Figure 5C). This was specifically the case for associations encoded during negative feedback (ρ = −0.476, *p* = 0.039; Figure 5D), but not for positive feedback (ρ = 0.250, *p* = 0.303; Figure 5E).

## Discussion

The present study addresses the influence of reward delivery and reward anticipation on associative memory formation. Participants performed an associative memory task where character-object associations were learned through positive and negative feedback. To investigate the influence of reward anticipation, different characters were associated with different positive vs. negative feedback ratios, while the influence of reward delivery was tested by comparing memory for associations encoded during positive vs. negative feedback. The memory for character-object associations were tested after a delay of either 20 min or 24 h, and the impact of individual sensitivity biases to reward and punishment was assessed via trait questionnaires. Critically, a computational approach was used to investigate the link between memory formation and subtle fluctuations in expected value and prediction error, two different aspects of reward known to be associated with the dopaminergic system. The results are discussed in detail below.

### Prediction error magnitude modulates the contribution of reward delivery to memory formation

Associative memory performance was better for character-object associations encoded during positive as compared to negative feedback, a result which extends previous studies reporting enhanced recognition memory for images associated with positive vs. negative feedback (Mather and Schoeke, 2011), and high vs. low monetary reward outcomes (Bialleck et al., 2011; Murayama and Kitagami, 2014).

Because the ability to discriminate between preferred and non-preferred objects in a character-object pair is equally dependent on the ability to encode positive, i.e., that an object is preferred, and negative associations, i.e., that an object is non-preferred, it might seem surprising that memory performance was better for positive as compared to negative associations. However, reward-related enhancements of memory formation have been attributed to the activation of the dopaminergic system and associated influences on hippocampal plasticity (Lisman and Grace, 2005; Shohamy and Adcock, 2010; Miendlarzewska et al., 2015). Dopamine neurons do not respond to an outcome unless it is more positive than predicted, i.e., positive prediction errors, or more negative than predicted, i.e., negative prediction errors, in which case there is a “dip” in activity (Schultz et al., 1997). Therefore, in the present study, learning positive associations may have benefitted from positive prediction errors causing increased dopaminergic influences on the hippocampus, while encoding negative associations may have suffered from reduced dopamine activity caused by negative prediction errors. Supporting this explanation, the computational approach showed significant and positive influences of prediction error magnitudes on memory performance, both when tested after 20 min and after 24 h. Additionally and importantly, we previously showed that the dopaminergic midbrain encodes the mismatch between actually receiving a happy smiley face and the expectation of receiving it, i.e., prediction errors (Aberg et al., 2015).

The finding that the prediction error magnitude during encoding correlated positively with memory performance when tested after a short delay, i.e., 20 min, is similar to recently obtained results (Davidow et al., 2016). Specifically, Davidow et al. (2016) showed that the prediction error magnitude positively influenced the incidental encoding of images, when memory was tested immediately after learning. Interestingly, this effect was found only in adolescents but not in adults. Because adolescents show a general increase in the activation of the dopaminergic reward system (Galvan, 2010; Van Leijenhorst et al., 2010; Davidow et al., 2016), as do adults with an increased expression of reward sensitive traits (Simon et al., 2010; Kennis et al., 2013), we predicted that individuals with an increased sensitivity for reward over punishment should display a stronger positive influence from prediction error magnitudes on subsequent memory performance, a prediction that was confirmed by the present results.

Our result that prediction error magnitudes correlated positively with memory performance when tested after 24 h contrasts with a recent study reporting a negative correlation between prediction error magnitude and memory performance for incidentally encoded images when memory was tested on the next day (Wimmer et al., 2014). Overnight delays between encoding and testing are likely to include sleep, thus offering an opportunity for sleep-related memory consolidation processes to occur (Diekelmann et al., 2009; Perogamvros and Schwartz, 2012; Rasch and Born, 2013; Igloi et al., 2015). One possible explanation for Wimmer et al. (2014)'s results is that consolidation processes enhanced and decremented memories encoded during negative and positive prediction errors, respectively. Furthermore, the activation of the dopaminergic reward system during sleep is important for memory consolidation (Perogamvros and Schwartz, 2012), and the degree of this activation is related to the expression of reward-related behaviors and traits during wakefulness (Perogamvros et al., 2012, 2015). Accordingly, consolidation processes dependent on the activation of the dopaminergic reward system could interact with individual traits to modulate memory formation during sleep. Supporting these speculations, we found that an increased sensitivity to reward over punishment was associated with increased memory for associations encoded during negative feedback when tested after 24 h, as compared to 20 min. Because some evidence suggests that sleep-related consolidation processes promote weakly encoded memories (Diekelmann et al., 2009; Oudiette et al., 2013; Rasch and Born, 2013), it is tempting to speculate that the memory encoding strength is directly related to the prediction error magnitude. Unfortunately, the present study was not designed to investigate sleep-related memory effects and further research is therefore needed to elucidate how individual traits influence reward-related memory processes during sleep. At the very least, the present results demonstrate the importance of considering individual traits when assessing reward-related memory enhancements.

Of note, other neurotransmitters such as noradrenalin and acetylcholine have also been implicated in learning and episodic memory formation (Doya, 2002; Harley, 2004; Tully and Bolshakov, 2010; Mather et al., 2016). Particularly relevant in the context of prediction errors is the noradrenergic system, with noradrenergic neurons in the locus coeruleus showing increased activity following events and outcomes that are either better or worse than predicted, thus encoding a notion of “surprise” or unexpected uncertainty (Harley, 2004; Yu and Dayan, 2005; Dayan, 2012; Clewett et al., 2014). Because noradrenergic neurons project to regions involved in memory formation and reward, including the hippocampus, the amygdala, and the dopaminergic midbrain, it could be predicted that the noradrenergic system enhances memory formation for any surprising event, and that the degree of surprise is determined by the magnitude of the unsigned prediction error. However, two computational models incorporating unsigned “noradrenergic” prediction errors provided inferior fits to behavioral data, as compared to a model incorporating signed “dopaminergic” prediction errors. Accordingly, the source of reward-related memory enhancements in the present study is more likely to be dopaminergic than noradrenergic.

### Reward anticipation determines memory formation through magnitude of expected value

Character-object associations for character types assigned to higher feedback ratios, i.e., more frequent positive vs. negative feedback, were better remembered as compared to those assigned to lower feedback ratios when tested 20 min, but not 24 h, after encoding.

Previous reports indicate enhanced memory formation for images signaling upcoming rewards (Wittmann et al., 2005; Bialleck et al., 2011), possibly due to increased reward anticipation (Adcock et al., 2006). While the present study did not test recognition memory for reward-predictive stimuli, i.e., the images of characters, the results are well in line with studies showing enhanced recognition-memory for other information presented in trials with high reward anticipation (Adcock et al., 2006; Spaniol et al., 2014).

The finding that memory performance was enhanced for character types associated with high feedback ratios after short, but not long time-delays, is in accordance with previous studies reporting effects of reward anticipation after short time-delays, i.e., up to 30 min (Bialleck et al., 2011; Mather and Schoeke, 2011), but contrasts with studies showing influences of reward anticipation on memory formation when memory is tested after long delays, i.e., >20 h (Wittmann et al., 2005, 2011, 2013; Murayama and Kitagami, 2014). While the reason for these rather discrepant results between studies are unclear, one explanation could be derived from the notion that dopamine plays an important role for memory consolidation processes (Lisman et al., 2011). Specifically, unlike most previous studies that used item recognition paradigms paired with monetary rewards, the present study investigated associative memory and provided happy or sad smiley faces as feedback. Thus, it could be suggested that, in most experimental contexts, monetary rewards may be more motivationally salient causing increased dopaminergic responses, which would then have a stronger impact on dopamine-dependent consolidation processes, ultimately leading to longer-lasting memory-benefits of reward anticipation (Shohamy and Adcock, 2010; Igloi et al., 2015).

Another explanation could be related to the fact that averaging memory performance across trials for specific reward conditions (such as different Character types in the present study) ignores the fine temporal distribution of delivery and anticipation of reward within and across conditions. In particular, the repeated pairing of a neutral cue, i.e., the face of a character type, with positive and rewarding outcomes causes a gradual increase in the reward predictive value of the cue, while conversely causing a gradual reduction of prediction errors at reward delivery. Indeed, using a computational approach that provides estimates of trial-wise fluctuations in these reward-related parameters, we demonstrate a significant influence of expected value on memory formation, both after 20 min as well as after 24 h, therefore indicating contributions of reward anticipation over both short and long delays. Further evidence supporting this explanation comes from robust findings showing that dopamine neuron activity does not reflect the absolute magnitude of reward, but instead tracks the magnitude of expected values and prediction errors. Specifically, reward-learning causes a shift in the dopaminergic response from the time of reward delivery to the presentation of a reward-predictive cue (Schultz et al., 1997). Thus, given that dopamine activity promotes memory formation (Lisman and Grace, 2005; Shohamy and Adcock, 2010; Lisman et al., 2011), a plausible speculation is that changes in memory encoding as a function of fluctuations in the magnitude of prediction error and expected value relate to similar fluctuations in the activity of the dopaminergic system. Given that the contributions of these very specific and important reward computations to memory formation have been largely ignored in the literature, the present results significantly extend previous research by providing a more accurate description of the link between reward, the dopamine system, and memory.

Notably, two recent studies used computational approaches to study the impact of reward anticipation on the incidental encoding of images during reinforcement learning tasks, but report no impact of reward anticipation, i.e., the expected value of a chosen alternative (Wimmer et al., 2014) or a reward-predictive cue (Davidow et al., 2016), on subsequent image recognition memory. One main difference between these studies and ours is that those previous studies looked at recognition memory while we used an associative memory paradigm. Moreover, we presented the associated objects together with the reward-predictive cue, i.e., the character face, during both the encoding and the test phase. Accordingly, it cannot be excluded that, besides its impact on memory encoding, the presence of a reward-predictive cue enhanced memory retrieval as well. Surprisingly few studies looked at the impact of reward and the dopamine system on memory retrieval. However, while memory retrieval is not enhanced by performance-based incentives alone (Han et al., 2010; Elward et al., 2015), the presentation of previously rewarded objects may contribute to the reinstatement of “non-strategic, reward-related processes” during memory retrieval (Kuhl et al., 2010; Halsband et al., 2012). Moreover, pattern completion, i.e., memory retrieval based on partial inputs, depends on intact dopamine function (Li et al., 2010). Additionally, presenting items associated with high (vs. low) rewards was found to engage the hippocampus and the dopaminergic midbrain during both encoding and retrieval (Wolosin et al., 2012). Together, these results suggest that previously rewarded objects may reinstate the reward context that was present during memory encoding, thus providing additional input which could enhance the memory retrieval process.

The present results may provide some insights concerning recent interesting findings by Mather and Schoeke (2011) showing that positive outcomes enhanced memory encoding also in subsequent trials. Specifically, a better than expected reward outcome (e.g., a positive feedback) may enable rewards to temporally extend their influence on memory formation by increasing expectations of rewards in subsequent trials. Notably, Mather and Schoeke (2011) found that this lingering effect of reward was significant for recognition memory but not when tested via free recall. It could be speculated that the absence of reward-related cues during free recall impeded memory retrieval processes by preventing the reinstatement of reward contexts. However, these results should be interpreted with caution due to the very low number of recalled items. More research is needed to further validate the lingering effects of reward on memory formation, and to disentangle the contribution of reward to memory encoding from its influence on retrieval processes.

Finally, most previous studies addressing the impact of reward on memory formation looked at item recognition memory or source memory. Here we show that anticipation and delivery of reward also enhances associative memory formation, thus extending previous results obtained via recognition memory paradigms. Altogether, these findings are in accordance with lesion studies suggesting that item recognition memory and associative memory depend on the same hippocampal neural circuitry (Stark and Squire, 2001; Stark et al., 2002), but see (Brown and Aggleton, 2001; Yonelinas et al., 2001).

### Limitations

The present behavioral results provide some first insights about the involvement of specific neurotransmitters in reward's impact on memory formation. As mentioned previously, dopamine, acetylcholine, and norepinephrine have all been linked to enhanced memory encoding (Doya, 2002; Harley, 2004; Lisman and Grace, 2005; Tully and Bolshakov, 2010; Lisman et al., 2011; Mather et al., 2016). Thus, to determine the involvement of specific neurotransmitter systems in reward-related memory formation, the computational approach outlined in the present study needs to be combined with pharmacology and/or neuroimaging.

Another limitation of the present study can be derived from recent evidence indicating significant gender differences in learning and memory (Herlitz et al., 1997; Piefke et al., 2005; Pauls et al., 2013), reward sensitivity (Chian-Shang et al., 2007; Byrne and Worthy, 2015), reward-related decision making (Mather and Lighthall, 2012), and dopamine function (Cosgrove et al., 2007; Riccardi et al., 2011). Because participants in the present study were mostly females, and information regarding their menstrual cycle was not obtained, the impact of gender or sex hormones cannot be accounted for here.

## Conclusion

Here we first use standard procedures, i.e., averaging memory performance across trials within conditions, to describe independent contributions to associative memory formation from reward delivery at short (20 min) and longer term (24 h), as well as from reward anticipation selectively at short term (20 min, but not when tested after 24 h). By contrast, a novel computational approach, which considers trial-by-trial fluctuations in the magnitude of prediction errors and expected values, revealed that reward delivery (as mediated by prediction error magnitude) and reward anticipation (as mediated by expected value magnitude) contribute to memory formation, both when tested after 20 min and after 24 h. Because this approach incorporates fundamental aspects of reward computations well-known to be reflected in dopamine neuron activity, it not only provides a more sensitive, but also a more accurate description of the link between reward, dopamine, and memory formation, than behavioral measures averaged across trials alone.

By clarifying how memory formation may be significantly modulated by distinct reward processing mechanisms and individual trait-dispositions, the present study also provides valuable insights for fields beyond basic neuroscience, such as for education or rehabilitation strategies. In particular, our findings highlight the importance of tailoring learning contexts on an individual basis, as well as the potential drawbacks when not doing so (as is the case in most of today's educational settings).

## Ethics statement

All participants provided written consent according to the ethical regulations of the Geneva University Hospital and the study was performed in accordance with the Declaration of Helsinki.

## Author contributions

KA, JM, and SS were involved in all aspects of the study from initial idea to submission of paper.

### Conflict of interest statement

The authors declare that the research was conducted in the absence of any commercial or financial relationships that could be construed as a potential conflict of interest.

## References

[B1] Aberg K. C. Doell K. C. Schwartz S. (2015). Hemispheric asymmetries in striatal reward responses relate to approach-avoidance learning and encoding of positive-negative prediction errors in dopaminergic midbrain regions. J. Neurosci. 35, 14491–14500. 10.1523/JNEUROSCI.1859-15.2015 26511241 PMC6605462

[B2] Aberg K. C. Doell K. C. Schwartz S. (2016). Linking individual learning styles to approach-avoidance motivational traits and computational aspects of reinforcement learning. PLoS ONE 11:e0166675 10.1371/journal.pone.0166675 27851807 PMC5113060

[B3] Adcock R. A. Thangavel A. Whitfield-Gabrieli S. Knutson B. Gabrieli J. D. (2006). Reward-motivated learning: mesolimbic activation precedes memory formation. Neuron 50, 507–517. 10.1016/j.neuron.2006.03.036 16675403

[B4] Akaike H. (1974). A new look at the statistical model identification. IEEE Trans. Automat. Contr. 19, 716–723. 10.1109/TAC.1974.1100705

[B5] Bialleck K. A. Schaal H. P. Kranz T. A. Fell J. Elger C. E. Axmacher N. (2011). Ventromedial prefrontal cortex activation is associated with memory formation for predictable rewards. PLoS ONE 6:e16695. 10.1371/journal.pone.0016695 21326612 PMC3033899

[B6] Brown M. W. Aggleton J. P. (2001). Recognition memory: what are the roles of the perirhinal cortex and hippocampus? Nat. Rev. Neurosci. 2, 51–61. 10.1038/35049064 11253359

[B7] Byrne K. A. Worthy D. A. (2015). Gender differences in reward sensitivity and information processing during decision-making. J. Risk Uncertain. 50, 55–71. 10.1007/s11166-015-9206-7

[B8] Chian-Shang R. L. Chen-Ying H. Wei-Yu L. Ching Wen V. C. (2007). Gender differences in punishment and reward sensitivity in a sample of Taiwanese college students. Pers. Individ. Dif. 43, 475–483. 10.1016/j.paid.2006.12.016

[B9] Clewett D. Schoeke A. Mather M. (2014). Locus coeruleus neuromodulation of memories encoded during negative or unexpected action outcomes. Neurobiol. Learn. Mem. 111, 65–70. 10.1016/j.nlm.2014.03.006 24667494 PMC4039187

[B10] Cosgrove K. P. Mazure C. M. Staley J. K. (2007). Evolving knowledge of sex differences in brain structure, function, and chemistry. Biol. Psychiatry 62, 847–855. 10.1016/j.biopsych.2007.03.001 17544382 PMC2711771

[B11] Davidow J. Y. Foerde K. Galvan A. Shohamy D. (2016). An upside to reward sensitivity: the hippocampus supports enhanced reinforcement learning in adolescence. Neuron 92, 93–99. 10.1016/j.neuron.2016.08.031 27710793

[B12] Dayan P. (2012). Twenty-five lessons from computational neuromodulation. Neuron 76, 240–256. 10.1016/j.neuron.2012.09.027 23040818

[B13] De Zubicaray G. I. Mcmahon K. L. Dennis S. Dunn J. C. (2011). Memory strength effects in fMRI studies: a matter of confidence. J. Cogn. Neurosci. 23, 2324–2335. 10.1162/jocn.2010.21601 21126157

[B14] Diekelmann S. Wilhelm I. Born J. (2009). The whats and whens of sleep-dependent memory consolidation. Sleep Med. Rev. 13, 309–321. 10.1016/j.smrv.2008.08.002 19251443

[B15] Doya K. (2002). Metalearning and neuromodulation. Neural Netw. 15, 495–506. 10.1016/S0893-6080(02)00044-8 12371507

[B16] Elward R. L. Vilberg K. L. Rugg M. D. (2015). Motivated memories: effects of reward and recollection in the core recollection network and beyond. Cereb. Cortex 25, 3159–3166. 10.1093/cercor/bhu109 24872520 PMC4537449

[B17] Gabrieli J. D. (1998). Cognitive neuroscience of human memory. Annu. Rev. Psychol. 49, 87–115. 10.1146/annurev.psych.49.1.87 9496622

[B18] Galvan A. (2010). Adolescent development of the reward system. Front. Hum. Neurosci. 4:6. 10.3389/neuro.09.006.2010 20179786 PMC2826184

[B19] Gold J. J. Hopkins R. O. Squire L. R. (2006). Single-item memory, associative memory, and the human hippocampus. Learn. Mem. 13, 644–649. 10.1101/lm.258406 16980546 PMC1635407

[B20] Halsband T. M. Ferdinand N. K. Bridger E. K. Mecklinger A. (2012). Monetary rewards influence retrieval orientations. Cogn. Affect. Behav. Neurosci. 12, 430–445. 10.3758/s13415-012-0093-y 22547161

[B21] Han S. Huettel S. A. Raposo A. Adcock R. A. Dobbins I. G. (2010). Functional significance of striatal responses during episodic decisions: recovery or goal attainment? J. Neurosci. 30, 4767–4775. 20357127 10.1523/JNEUROSCI.3077-09.2010 PMC3433049

[B22] Harley C. W. (2004). Norepinephrine and dopamine as learning signals. Neural Plast. 11, 191–204. 10.1155/NP.2004.191 15656268 PMC2567044

[B23] Herlitz A. Nilsson L. G. Backman L. (1997). Gender differences in episodic memory. Mem. Cognit. 25, 801–811. 10.3758/BF03211324 9421566

[B24] Howell D. (2013). Statistical Methods for Psychology. Belmont, CA: Wadsworth.

[B25] Igloi K. Gaggioni G. Sterpenich V. Schwartz S. (2015). A nap to recap or how reward regulates hippocampal-prefrontal memory networks during daytime sleep in humans. Elife 4:e07903. 10.7554/eLife.07903 26473618 PMC4721959

[B26] Kennis M. Rademaker A. R. Geuze E. (2013). Neural correlates of personality: an integrative review. Neurosci. Biobehav. Rev. 37, 73–95. 10.1016/j.neubiorev.2012.10.012 23142157

[B27] Kensinger E. A. (2004). Remembering emotional experiences: the contribution of valence and arousal. Rev. Neurosci. 15, 241–251. 10.1515/REVNEURO.2004.15.4.241 15526549

[B28] Kuchinke L. Fritzemeier S. Hofmann M. J. Jacobs A. M. (2013). Neural correlates of episodic memory: associative memory and confidence drive hippocampus activations. Behav. Brain Res. 254, 92–101. 10.1016/j.bbr.2013.04.035 23644182

[B29] Kuhl B. A. Shah A. T. Dubrow S. Wagner A. D. (2010). Resistance to forgetting associated with hippocampus-mediated reactivation during new learning. Nat. Neurosci. 13, 501–506. 10.1038/nn.2498 20190745 PMC2847013

[B30] Lansink C. S. Goltstein P. M. Lankelma J. V. Mcnaughton B. L. Pennartz C. M. (2009). Hippocampus leads ventral striatum in replay of place-reward information. PLoS Biol. 7:e1000173. 10.1371/journal.pbio.1000173 19688032 PMC2717326

[B31] Lardi C. Billieux J. D'acremont M. Van Der Linden M. (2008). A french adaptation of a short version of the sensitivity to punishment and sensitivity to reward questionnaire (SPSRQ). Pers. Individ. Dif. 45, 722–725. 10.1016/j.paid.2008.07.019

[B32] Li F. Wang L. P. Shen X. M. Tsien J. Z. (2010). Balanced dopamine is critical for pattern completion during associative memory recall. PLoS ONE 5:e15401. 10.1371/journal.pone.0015401 21060884 PMC2965183

[B33] Lisman J. E. Grace A. A. (2005). The hippocampal-VTA loop: controlling the entry of information into long-term memory. Neuron 46, 703–713. 10.1016/j.neuron.2005.05.002 15924857

[B34] Lisman J. Grace A. A. Duzel E. (2011). A neoHebbian framework for episodic memory; role of dopamine-dependent late LTP. Trends Neurosci. 34, 536–547. 10.1016/j.tins.2011.07.006 21851992 PMC3183413

[B35] Maren S. Quirk G. J. (2004). Neuronal signalling of fear memory. Nat. Rev. Neurosci. 5, 844–852. 10.1038/nrn1535 15496862

[B36] Mather M. Clewett D. Sakaki M. Harley C. W. (2016). Norepinephrine ignites local hotspots of neuronal excitation: how arousal amplifies selectivity in perception and memory. Behav. Brain Sci. 39:e200 10.1017/S0140525X15000667 PMC5830137 26126507

[B37] Mather M. Lighthall N. R. (2012). Both Risk and Reward are processed differently in decisions made under stress. Curr. Dir. Psychol. Sci. 21, 36–41. 10.1177/0963721411429452 22457564 PMC3312579

[B38] Mather M. Schoeke A. (2011). Positive outcomes enhance incidental learning for both younger and older adults. Front. Neurosci. 5:129. 10.3389/fnins.2011.00129 22125509 PMC3221314

[B39] Mayes A. Montaldi D. Migo E. (2007). Associative memory and the medial temporal lobes. Trends Cogn. Sci. 11, 126–135. 10.1016/j.tics.2006.12.003 17270487

[B40] Miendlarzewska E. A. Bavelier D. Schwartz S. (2015). Influence of reward motivation on human declarative memory. Neurosci. Biobehav. Rev. 61, 156–176. 10.1016/j.neubiorev.2015.11.015 26657967

[B41] Murayama K. Kitagami S. (2014). Consolidation power of extrinsic rewards: reward cues enhance long-term memory for irrelevant past events. J. Exp. Psychol. Gen. 143, 15–20. 10.1037/a0031992 23421444

[B42] Nelder J. A. Mead R. (1965). A simplex method for function minimization. Comput. J. 7, 308–313. 10.1093/comjnl/7.4.308

[B43] Oudiette D. Antony J. W. Creery J. D. Paller K. A. (2013). The role of memory reactivation during wakefulness and sleep in determining which memories endure. J. Neurosci. 33, 6672–6678. 10.1523/JNEUROSCI.5497-12.2013 23575863 PMC3677604

[B44] Pauls F. Petermann F. Lepach A. C. (2013). Gender differences in episodic memory and visual working memory including the effects of age. Memory 21, 857–874. 10.1080/09658211.2013.765892 23383629

[B45] Perogamvros L. Aberg K. Gex-Fabry M. Perrig S. Cloninger C. R. Schwartz S. (2015). Increased reward-related behaviors during sleep and wakefulness in sleepwalking and idiopathic nightmares. PLoS ONE 10:e0134504. 10.1371/journal.pone.0134504 26287974 PMC4546110

[B46] Perogamvros L. Baud P. Hasler R. Cloninger C. R. Schwartz S. Perrig S. (2012). Active reward processing during human sleep: insights from sleep-related eating disorder. Front. Neurol. 3:168. 10.3389/fneur.2012.00168 23205019 PMC3506891

[B47] Perogamvros L. Schwartz S. (2012). The roles of the reward system in sleep and dreaming. Neurosci. Biobehav. Rev. 36, 1934–1951. 10.1016/j.neubiorev.2012.05.010 22669078

[B48] Piefke M. Weiss P. H. Markowitsch H. J. Fink G. R. (2005). Gender differences in the functional neuroanatomy of emotional episodic autobiographical memory. Hum. Brain Mapp. 24, 313–324. 10.1002/hbm.20092 15704151 PMC6871670

[B49] Qin S. Van Marle H. J. Hermans E. J. Fernandez G. (2011). Subjective sense of memory strength and the objective amount of information accurately remembered are related to distinct neural correlates at encoding. J. Neurosci. 31, 8920–8927. 10.1523/JNEUROSCI.2587-10.2011 21677175 PMC6622961

[B50] Rasch B. Born J. (2013). About sleep's role in memory. Physiol. Rev. 93, 681–766. 10.1152/physrev.00032.2012 23589831 PMC3768102

[B51] Rescorla R. Wagner A. (1972). A Theory of Pavlovian Conditioning: Variations in the Effectiveness of Reinforcement and Non Reinforcement. New York, NY: Appleton-Century-Crofts.

[B52] Riccardi P. Park S. Anderson S. Doop M. Ansari M. S. Schmidt D. (2011). Sex differences in the relationship of regional dopamine release to affect and cognitive function in striatal and extrastriatal regions using positron emission tomography and [(1)(8)F]fallypride. Synapse 65, 99–102. 10.1002/syn.20822 20506565 PMC2965297

[B53] Schultz W. Dayan P. Montague P. R. (1997). A neural substrate of prediction and reward. Science 275, 1593–1599. 10.1126/science.275.5306.1593 9054347

[B54] Schultz W. Dickinson A. (2000). Neuronal coding of prediction errors. Annu. Rev. Neurosci. 23, 473–500. 10.1146/annurev.neuro.23.1.473 10845072

[B55] Shohamy D. Adcock R. A. (2010). Dopamine and adaptive memory. Trends Cogn. Sci. 14, 464–472. 10.1016/j.tics.2010.08.002 20829095

[B56] Simon J. J. Walther S. Fiebach C. J. Friederich H. C. Stippich C. Weisbrod M.. (2010). Neural reward processing is modulated by approach- and avoidance-related personality traits. Neuroimage 49, 1868–1874. 10.1016/j.neuroimage.2009.09.016 19770056

[B57] Smillie L. D. Dalgleish L. I. Jackson C. J. (2007). Distinguishing between learning and motivation in behavioral tests of the reinforcement sensitivity theory of personality. Pers. Soc. Psychol. Bull. 33, 476–489. 10.1177/0146167206296951 17363762

[B58] Spaniol J. Schain C. Bowen H. J. (2014). Reward-enhanced memory in younger and older adults. J. Gerontol. B Psychol. Sci. Soc. Sci. 69, 730–740. 10.1093/geronb/gbt044 23690000

[B59] Squire L. R. Stark C. E. Clark R. E. (2004). The medial temporal lobe. Annu. Rev. Neurosci. 27, 279–306. 10.1146/annurev.neuro.27.070203.144130 15217334

[B60] Stark C. E. L. Bayley P. J. Squire L. R. (2002). Recognition memory for single items and for associations is similarly impaired following damage to the hippocampal region. Learn. Mem. 9, 238–242. 10.1101/lm.51802 12359833 PMC187132

[B61] Stark C. E. Squire L. R. (2001). Simple and associative recognition memory in the hippocampal region. Learn. Mem. 8, 190–197. 10.1101/lm.40701 11533222 PMC311379

[B62] Tobler P. N. Fiorillo C. D. Schultz W. (2005). Adaptive coding of reward value by dopamine neurons. Science 307, 1642–1645. 10.1126/science.1105370 15761155

[B63] Tully K. Bolshakov V. Y. (2010). Emotional enhancement of memory: how norepinephrine enables synaptic plasticity. Mol. Brain 3:15. 10.1186/1756-6606-3-15 20465834 PMC2877027

[B64] Van Leijenhorst L. Zanolie K. Van Meel C. S. Westenberg P. M. Rombouts S. A. Crone E. A. (2010). What motivates the adolescent? brain regions mediating reward sensitivity across adolescence. Cereb. Cortex 20, 61–69. 10.1093/cercor/bhp078 19406906

[B65] Watkins C. Dayan P. (1992). Q-learning. Mach. Learn. 279–292. 10.1007/BF00992698

[B66] Wimmer G. E. Braun E. K. Daw N. D. Shohamy D. (2014). Episodic memory encoding interferes with reward learning and decreases striatal prediction errors. J. Neurosci. 34, 14901–14912. 10.1523/JNEUROSCI.0204-14.2014 25378157 PMC4220024

[B67] Wittmann B. C. Dolan R. J. Duzel E. (2011). Behavioral specifications of reward-associated long-term memory enhancement in humans. Learn. Mem. 18, 296–300. 10.1101/lm.1996811 21502336 PMC3465832

[B68] Wittmann B. C. Schott B. H. Guderian S. Frey J. U. Heinze H. J. Duzel E. (2005). Reward-related FMRI activation of dopaminergic midbrain is associated with enhanced hippocampus-dependent long-term memory formation. Neuron 45, 459–467. 10.1016/j.neuron.2005.01.010 15694331

[B69] Wittmann B. C. Tan G. C. Lisman J. E. Dolan R. J. Duzel E. (2013). DAT genotype modulates striatal processing and long-term memory for items associated with reward and punishment. Neuropsychologia 51, 2184–2193. 10.1016/j.neuropsychologia.2013.07.018 23911780 PMC3809516

[B70] Wolosin S. M. Zeithamova D. Preston A. R. (2012). Reward modulation of hippocampal subfield activation during successful associative encoding and retrieval. J. Cogn. Neurosci. 24, 1532–1547. 10.1162/jocn_a_00237 22524296 PMC3393089

[B71] Yonelinas A. P. Hopfinger J. B. Buonocore M. H. Kroll N. E. Baynes K. (2001). Hippocampal, parahippocampal and occipital-temporal contributions to associative and item recognition memory: an fMRI study. Neuroreport 12, 359–363. 10.1097/00001756-200102120-00035 11209950

[B72] Yu A. J. Dayan P. (2005). Uncertainty, neuromodulation, and attention. Neuron 46, 681–692. 10.1016/j.neuron.2005.04.026 15944135

